# Development of Seawater Reverse Osmosis Configurations for Low- and High-Fouling Feedwaters: A Techno-Economic Review of FilmTec Membranes Performance

**DOI:** 10.3390/membranes16040149

**Published:** 2026-04-16

**Authors:** Antonio Casañas Gonzalez, Federico Antonio Leon Zerpa, Alejandro Ramos Martin

**Affiliations:** 1 Dow Water and Process Solutions, Dow Ibérica S.L., 35017 Las Palmas de Gran Canaria, Spain; tonicasanas@hotmail.com; 2 Process Engineering Department, University of Las Palmas de Gran Canaria, 35017 Las Palmas de Gran Canaria, Spain; alejandro.ramos@ulpgc.es

**Keywords:** membrane, seawater, desalination, biofoluing, CIP, system-design

## Abstract

This work presents the most recent advancements and operational experiences obtained with the large-active-area, high-rejection FilmTec™ SW30HR-380 and SW30HR-320 reverse osmosis membrane elements, with particular focus on their techno-economic implications, especially regarding energy demand and potential operational cost reductions. The study also examines fouling prevalence and reviews the latest developments in technical mitigation strategies, with emphasis on the new wide-spacer SW30HR-320 elements designed for open-intake applications. Overall, the findings indicate that these new membrane products constitute an effective option for the design of seawater reverse osmosis systems treating both clean and fouling-prone feedwaters. The techno-economic evaluation demonstrates that the adoption of these elements can enable reductions of approximately 20% in capital expenditures, up to 25% in energy consumption, and up to 4% in cleaning-related costs—including downtime—when the SW30HR-320 is operated under high-fouling feedwater conditions.

## 1. Introduction

The origins of membrane use are not precisely documented, and it remains unclear when these materials were first applied in practical separation processes. Nevertheless, it is widely acknowledged that the conceptual foundations of membrane science can be traced back to the eighteenth century. A pivotal moment occurred in 1748, when Abbé Nollet reported experiments demonstrating selective transport across natural membranes, notably through the concentration of alcohol from aqueous mixtures. These early observations established the fundamental concept of semipermeability, which would later underpin the development of membrane-based separation technologies [1,2].

Significant theoretical and experimental advances followed sporadically over the next two centuries, culminating in the mid-twentieth century with the emergence of reverse osmosis (RO) as a viable industrial process. In 1959, Reid and Breton made a major breakthrough with the development of cellulose acetate membranes capable of desalinating brackish water under applied pressure. Shortly thereafter, in 1962, Loeb and Sourirajan introduced the asymmetric membrane structure, characterized by a thin selective skin supported by a porous sublayer. This innovation dramatically improved water permeability while maintaining adequate salt rejection, marking the birth of modern RO membrane technology. These early cellulosic materials thus represented the first synthetic membranes to achieve widespread industrial relevance and commercial adoption in water treatment applications [1,3,4].

Despite their success, cellulose acetate membranes exhibited several inherent limitations, including sensitivity to hydrolysis, restricted operating pH ranges, moderate salt rejection, and vulnerability to biological degradation. These constraints motivated intensive research efforts aimed at developing alternative membrane materials with superior performance and durability. A major leap forward occurred during the 1970s through the pioneering work of J. Cadotte and collaborators, who developed the first commercially successful thin-film composite (TFC) polyamide membrane via interfacial polymerization. Initially applied in brackish water desalination, TFC membranes soon demonstrated clear advantages over cellulose acetate technology [1,5,6].

Compared with earlier cellulose acetate membranes—which had been formulated in the late 1950s and commercially deployed by the early 1960s—polyamide TFC membranes provided substantially higher salt rejection, improved chemical stability, enhanced mechanical robustness, and significantly greater water permeability at lower operating pressures. These attributes translated into higher productivity per unit membrane area and lower specific energy consumption, ultimately reducing both capital and operating costs. As a result, polyamide-based TFC membranes rapidly became the dominant technology within the expanding reverse osmosis industry, effectively establishing the material platform still used in most RO systems today [1,7,8].

The rapid progress in polymer science, materials engineering, and membrane manufacturing during the 1980s further accelerated these developments. Supported by industrial stakeholders with strong technological and fabrication capabilities, TFC-PA membrane technology was continuously refined, leading to improvements in membrane chemistry, module design, and element configuration. This momentum enabled the widespread deployment of RO systems for seawater desalination, extending membrane applications beyond brackish water into high-salinity environments. Consequently, seawater reverse osmosis (SWRO) plants evolved from small-scale installations producing a few thousand cubic meters per day into large industrial facilities with capacities exceeding 50,000 m^3^/d, and eventually into today’s mega-scale desalination plants [9,10,11].

These advancements laid the groundwork for ongoing innovations in high-rejection, high-productivity membrane elements optimized for energy efficiency, fouling resistance, and long-term operational stability—key challenges that continue to shape modern SWRO system design and operation [12].

## 2. Design Evolution and Initial Field Deployment of the Seawater SW30HR FilmTec Membranes

### 2.1. Design Evolution and Initial Field Deployment of the Seawater SW30HR-380 FilmTec Membranes

The first installations of the SW30HR-380 membrane elements were carried out in 1993, where early field evaluations demonstrated excellent operational behavior. Testing continued through 1994 with consistently positive performance and no reported failures. From 1995 onward, the element saw increasing deployment across multiple industrial desalination plants. It has since been marketed commercially under the name FilmTec SW30HR-380 (FilmTec, Edina, MN, USA) [13,14,15,16,17,18]. Its strong operational record has been supported by detailed studies describing both economic and environmental benefits associated with its use [19]. In the sections that follow, the configuration and selected performance data from the Ceuta seawater RO facility employing this membrane are presented.

### 2.2. Design Evolution and Initial Field Deployment of the Seawater FilmTec SW30HR-320 Membranes

During 1996, FilmTec Corporation (Edina, MN, USA) introduced a redesigned seawater reverse osmosis element intended for use in SWRO facilities that rely on open-ocean intakes and other feedwaters prone to elevated fouling levels. This updated model incorporated the widest feed spacer then available for water treatment applications—34 mil—which was selected to enhance flow dynamics and reduce particle accumulation. The design strategy capitalized on Dow’s advanced element-manufacturing methods, enabling a larger active membrane surface to be integrated into a standard 8-inch element without altering its external dimensions. By pairing this expanded surface area with the thicker spacer, the resulting element achieved a membrane area of approximately 320 ft^2^—greater than that of the SW30HR-8040 but somewhat lower than the SW30HR-380—while maintaining a stable hydraulic balance and increasing flow turbulence through its specialized spacer geometry. These modifications collectively lowered fouling susceptibility and improved the ease of chemical cleaning. Overall, the new configuration delivered about a 25% increase in permeate production when compared with the conventional SW30HR-8040 design. A portion of this gain, specifically the rise from 4000 to 5000 GPD (630–790 L/h) under standard test conditions, can be attributed to the increase in active area from 299 to 320 ft^2^ (27.9 to 30.0 m^2^) [20,21,22,23].

A second factor contributing to the improved performance of these FilmTec models was an updated internal architecture made possible by the novel fabrication approach. This advancement raised the flux efficiency of the company’s established thin-film composite technology from roughly 80% to essentially 100% in both the SW30HR-380 and SW30HR-320 elements. Flux efficiency compares permeate output measured from flat-sheet membrane samples under laboratory testing to the performance of the same membrane once integrated into a spiral-wound element; achieving 100% indicates that performance remains unchanged between the two configurations [24,25,26,27].

A detailed analysis of how these new designs differ from conventional 8-inch membrane elements available on the market can be found in [8,10]. Specifications for the SW30HR-380 and SW30HR-320 seawater elements appear in Table 1, showing performance improvements of approximately 50% and 25%, respectively, relative to the earlier SW30HR-8040 model, along with a reduction in salt passage of around 34% for these high-rejection versions [28,29,30].

## 3. Field Experiences Using SW30HR-380 Filmtec Membranes

The strong operational results demonstrated by the FilmTec SW30HR-380 membrane led to its adoption in numerous commercial seawater reverse osmosis (SWRO) installations, where it consistently delivered the required permeate flow and quality with outstanding reliability. The following case study examines the technical and economic aspects of its use, with particular emphasis on potential reductions in capital expenditure and operating costs. Improvements in energy performance represent the most influential factor in lowering total operating expenses [31,32,33].

### 3.1. Case History No. 1: Field Experiences Using SW30HR-380 Filmtec Membranes

This case study describes the performance of an SWRO facility located in a Spanish territory in Northern Africa. The plant was designed, built, and is currently operated by a joint venture between the Spanish engineering firms Pridesa and Cadagua, both headquartered in Bilbao. The installation is composed of three parallel trains, each consisting of 90 pressure vessels arranged in a single stage, with six elements per vessel—resulting in a total of 540 elements per train and 1620 FilmTec SW30HR-380 elements across the entire facility.

Feedwater is extracted from shallow coastal wells and subsequently treated through a sequence of chlorination, sand filtration, dechlorination, and cartridge filtration before being pressurized and delivered to the SWRO trains. This pretreatment sequence significantly improves water quality, reducing the silt density index (SDI15) from 3.8 to levels below measurable limits, while delivering an RO feed with an SDI05 between 2.9 and 5.0.

Feedwater temperature in the plant fluctuates between 17 and 21 °C, and the salt content (TDS) ranges from 37,800 to 39,150 ppm depending on local ocean currents. This variation reflects the mixing of waters from the Atlantic Ocean and the Mediterranean Sea, with the latter accounting for the higher salinity peaks.

The first of the three trains began operation in June 1998, with the remaining two following over the next six months. Table 2 presents representative operational data; Train B is shown as an example, although all three trains exhibit comparable behavior.

The fouling factor (FF) is a dimensionless parameter used to quantify the extent of hydraulic performance deterioration of a reverse osmosis membrane due to fouling and compaction. It reflects the deviation of actual operating conditions from clean-membrane reference conditions by comparing measured permeate flow to the expected permeate flow under identical normalized conditions.

A fouling factor lower than unity indicates performance loss caused by reversible and irreversible fouling phenomena.

Formula:

FF = Q_measured,norm_/Q_reference_

where

Q_measured,norm_ is the measured permeate flow normalized to reference conditions,

Q_reference_ is the reference permeate flow of a clean membrane under the same normalized conditions.

In some operational practices, the fouling factor is expressed inversely as a resistance-based indicator; however, the formulation above is consistent with the usage adopted in this study and in most RO system performance evaluations.

The normalized permeate flow is the permeate flow rate corrected to standard reference conditions of temperature, pressure, salinity, and recovery. Normalization removes the influence of variable operating conditions, allowing a direct and objective assessment of membrane fouling, compaction, and aging over time.

Normalized flow is a key performance indicator for tracking membrane health and long-term degradation.

Formula

Q_norm_ = Q_measured_ × TCF × (ΔP_ref_ − Δπ_ref_)/(ΔP_measured_ − Δπ_measured_)

where:

Q_norm_ = normalized permeate flow

Q_measured_ = measured permeate flow

TCF = temperature correction factor

ΔP transmembrane hydraulic pressure difference

Δπ = osmotic pressure difference

subscript ref refers to reference test conditions

subscript measured refers to actual operating conditions

The normalized salt rejection represents the salt rejection performance of a membrane corrected to reference operating conditions. Normalization eliminates the influence of temperature and recovery effects, enabling accurate comparison of intrinsic membrane selectivity over time.

It is particularly useful for detecting chemical degradation, mechanical damage, or excessive membrane aging.

Formula, salt rejection is defined as:SR = [1 − (Cp/Cf)] × 100

where:

Cp = permeate salt concentration

Cf = feed salt concentration

The normalized salt rejection is then expressed as:

SR = [1 − (Cp,norm,/Cf,norm)] × 100

with permeate and feed concentrations corrected to reference conditions using standard RO normalization procedures.

### 3.2. Energy Use of the HR-380 and Comparative Operating Cost Reductions Relative to Standard Elements

A comparable facility operating with standard membrane elements would require close to 700 elements per train to achieve the same average permeate production. In addition, such a configuration would need approximately 5.3% more feed pressure and would consume around 0.18 kWh per cubic meter of permeate above the consumption of the Ceuta installation. Over the course of a month, this additional demand corresponds to roughly 30,000 kWh of extra energy use. When these higher operational costs are contrasted with the reduced fouling and improved cleanability associated with the SW30HR-380 element, the resulting economic benefit is substantial—about USD 34,000 per train each year, or nearly USD 100,000 annually for the entire plant.

### 3.3. Efficiency of CIP

Cleaning performance using a conventional alkaline protocol, initiated once the hydraulic fouling index reached 0.87, proved to be relatively effective, yielding an increase in normalized permeate flow of around 14%. Regarding energy consumption, the system in a freshly cleaned state required approximately 3.42 kWh/m^3^, compared with 3.52 kWh/m^3^ after roughly three months of operation post-cleaning.

This 0.1 kWh/m^3^ difference translates into an energy saving of about 16,000 kWh during the first month following chemical cleaning for a single train. At an electricity cost of 8 US $/kWh, this corresponds to roughly USD 1300 saved in that same period.

When extrapolated to annual operation for the entire facility, total savings fall within the range of USD 100,000 to USD 160,000, depending on cleaning frequency and operational conditions. If the plant were equipped with conventional membrane elements instead, these savings would be reduced by approximately 36%.

## 4. Operational Insights from Middle Eastern Installations with the FilmTec SW30HR-380 Membranes

### 4.1. Case History No. 2: Commercial Experience in Field in Middle Eastern Installations with Open Intake

This case study concerns an SWRO installation located in the Middle East, an area where similar facilities have encountered operational challenges, primarily related to significant fouling. The water source is a direct surface intake exposed to environmental variability.

The raw feedwater entering the pretreatment system can exhibit total dissolved solids (TDS) as high as 47,000 ppm. Dosing with chlorine, ferric chloride, and sodium bisulfite is applied before sand filtration and cartridge filtration, after which the water is delivered to the RO unit. During operation, the plant typically receives feedwater with TDS values between 46,700 and 47,000 mg/L, an SDI_15_ in the range of 2.8 to 3.2, and temperatures fluctuating from 19 to 37 °C, most commonly near 22 °C.

The plant’s design required each pressure vessel to deliver between 3.5 and 3.6 m^3^/h of permeate, with product water salinity below 400 mg/L. Across all installations to date, the SW30HR-380 element has consistently met or exceeded expectations, often producing at least 15% more permeate than the nominal specification. While the original projection assumed a hydraulic fouling factor of 1.15 and an operating pressure of approximately 72.5 bar, actual operation has shown better performance: permeate quality surpasses the projections, and the feed pressure has been measured at 68.7 bar.

The observed average permeate flux is approximately 17.0 L/m^2^·h, with a system recovery of 36% in a single-stage configuration of six elements per pressure vessel. Table 3 summarizes the performance parameters for one representative vessel. The facility operates intermittently rather than in continuous mode.

The facility operates without any energy-recovery system. Under these conditions, and with a brine-side osmotic pressure of 54.3 bar, its measured energy demand reaches 6.6 kWh per cubic meter of produced permeate.

Table 4 presents a comparison between the plant’s current specific energy use and the theoretically achievable values when employing the FilmTec SW30HR-380 membrane, as well as a standard high-rejection seawater membrane. This comparison, based on the plant’s fixed recovery rate, illustrates how the unit could be run and what level of energy savings could be achieved under Middle Eastern operating conditions when substituting the conventional element with the newer one.

Only the energy associated with the high-pressure pump and the booster has been considered. To produce the same permeate output at this recovery rate, a conventional 5000 GPD spiral-wound element would require approximately 61% more active membrane surface. This estimate assumes—optimistically—that after four months of operation with a fouling-prone feed, the hydraulic fouling factor remains at 0.9.

The attainable specific energy demand is calculated using the pump and energy-recovery turbine efficiencies listed in Table 4.

Under the range of operating conditions examined in this case study, adopting the FilmTec SW30HR-380 elements instead of the standard 5000 GPD spirals yields energy savings of roughly 20.8–22.6%. Beyond the sizeable potential capital reduction for the RO section (around 60%) and the 21.8% decrease in energy usage, the key implication is that seawater desalination at high salinity can be achieved at acceptable costs without increasing the feed pressure—an action that often triggers several challenges. Raising the feed pressure, for instance, elevates energy expenditure due to higher pumping power requirements. Moreover, higher flow rates through high-flux membranes accelerate fouling, shorten membrane life, and increase the frequency and cost of cleaning, replacement, and associated maintenance. Operating closer to equipment limits also results in faster wear of pumps, valves, and other components.

In summary, the enhanced internal configuration and expanded active surface area of the FilmTec elements deliver higher productivity without relying on high-flux membranes or elevated feed pressures—conditions that would otherwise raise energy costs, intensify fouling, and increase the frequency and financial burden of membrane replacement and disposal.

### 4.2. Case History No. 3: Middle East Installation with Seawell Intake, Elevated Temperature, and Partial Second Pass

This case study describes a desalination facility in the Middle East operating at 32 °C, sourcing seawater from a coastal well. Pretreatment is straightforward because the well water quality is relatively good. The process includes multimedia filtration followed by 5-micron cartridges, with no chlorine dosing; sodium metabisulfite is added at the well outlet.

The feed salinity is 45,400 ppm, and the system incorporates a partial second pass to obtain a blended product below 300 ppm. Specifically, 15 m^3^/h of second-pass permeate (11 ppm) is mixed with 30 m^3^/h of first-pass permeate (364 ppm).

Performance results are shown in Table 5. The plant has run for six months without operational issues, with specific energy consumptions of 3.7 kWh/m^3^ for the first pass and 0.55 kWh/m^3^ for the second pass. Since only one-third of the first-pass permeate undergoes a second pass, the combined specific energy consumption for the blended permeate—at roughly 290 ppm TDS—is approximately 3.9 kWh/m^3^. The system has not yet required chemical cleaning, and no cleaning events are anticipated during the first year of operation.

## 5. Field Performance of FilmTec SW30HR-320

### 5.1. Case History No. 4: Operation with FilmTec SW30HR-320 at a Site with Open Water Intake

FilmTec SW30HR-320 elements have been deployed at multiple locations. This case report summarizes the results from a system commissioned in July 1997. Prior to installing the new elements, the facility experienced operational difficulties when using conventional membrane modules.

The permeate TDS initially decreased to 360 ppm after roughly 140 h of operation but later rose to 429 ppm as a result of mechanical leakage in one of the pressure vessels. The efficiencies of the high-pressure pump and the energy-recovery turbine are 0.84 and 0.87, respectively.

The system draws water from an open sea intake where the raw water quality is poor. The feed’s SDI(15) ranges between 2.4 and 4.6, with values exceeding 3.8 for about 30% of the operating period. The source water contains significant levels of colloidal material and biological contamination, and the total bacterial count varies from 300 CFU/mL to TNTC (“too numerous to count”).

Pretreatment includes acid dosing, chlorination, two-stage sand filtration, dechlorination, and cartridge filtration. After this, the RO feed is delivered to the membrane system by a high-pressure pump (HPP) characterized by a steep pressure rise—from 3 bar to 65 bar in 16 s. The installation comprises 24 pressure vessels, each loaded with seven membrane elements.

The combination of poor feedwater quality and the very rapid pressurization from the HPP created considerable mechanical stress on elements designed with conventional spacers. As a consequence, several elements sustained mechanical damage. The average interval between chemical cleanings dropped dramatically—from around seven weeks to only between ten and fifteen days.

Under these operating conditions, SW30HR-320 membrane modules were installed in several pressure vessels, while a separate vessel was equipped with newly manufactured conventional membranes to provide a baseline for evaluation. Before resuming production, the entire desalination facility was subjected to an exhaustive cleaning process, after which operation was restarted using the updated membrane configuration.

The comparative assessment is reported in Table 6 based on normalized performance indicators. This approach was required because the system continued operating while still containing a limited number of previously installed membrane elements.

### 5.2. Fouling Resistance and Cleanability of the FilmTec SW30HR-320

The performance of the newly implemented membrane demonstrates a marked improvement in resistance to fouling. When subjected to equivalent fouling loads in the incoming feedwater, the SW30HR-320 exhibited a capacity reduction of 17% after the first year of operation and 20.5% after two years. By contrast, the conventional membranes showed more pronounced performance deterioration, with capacity losses of 23.5% after one year and 31.3% after two years of service.

These results indicate that the decline in permeate flow experienced by the SW30HR-320 is approximately 35% lower than that observed for the conventional membranes—representing a reduction of more than one third and highlighting a substantial operational advantage. Moreover, the cleanability of the FilmTec SW30HR-320 is particularly notable, with superior recovery performance following cleaning procedures, as illustrated by the data summarized in Table 7.

Start-up measurements were taken 78 h after commissioning. The one-year performance data correspond to readings collected 354 days after start-up and 88 h after an alkaline cleaning procedure. The two-year data set was recorded 739 h after start-up and 59 h following alkaline cleaning.

All of the remaining conventional elements were eventually replaced with new SW30HR-320 modules; therefore, no performance data are available for the older units thereafter. Salt rejection values were determined based on chloride concentration analysis.

The stabilized hydraulic fouling factor (FF_st_) is measured after the rapid initial decline in transient permeate flow has ended—typically around 500 h (≈20 days) after start-up. At SWRO Site 37, this parameter was 1.02 for the HR-320 elements and 0.99 for the conventional ones.

Long-term operational data collected from open-intake SWRO installations demonstrate that the wide-spacer geometry of the SW30HR-320 elements plays a decisive role in sustaining flux recovery over multiple cleaning-in-place (CIP) cycles. As shown by the two-year monitoring dataset, HR-320 membranes consistently exhibited higher normalized permeate flow recovery following alkaline cleanings compared to conventional elements, with average flow restoration in the range of 17–19% after a single CIP cycle, even after prolonged operation under elevated fouling loads. In contrast, conventional membranes required multiple cleaning stages to achieve lower recovery levels (typically 9–12%), indicating a higher degree of irreversible fouling accumulation. Post-operation inspections and performance trends suggest that the enlarged feed-spacer thickness (34 mil) enhances local turbulence and reduces stagnant zones, thereby facilitating the detachment and removal of compressible biological fouling layers, which are predominant in open sea intake systems. At the same time, the wider flow channels mitigate the compaction and wedging of inorganic and colloidal foulants, resulting in lower pressure-drop buildup and improved cleanability. These effects are reflected in the significantly slower long-term decline of normalized permeate flow and the reduced frequency and intensity of required cleaning events. Collectively, the CIP recovery behavior and sustained hydraulic performance confirm that feed-spacer geometry is a critical design parameter governing fouling reversibility and long-term operational stability in SWRO systems subject to biologically and particulate-rich feedwaters.

### 5.3. Energy Use of HR-320 and Cost Advantages Compared with Conventional Elements

The plant equipped with traditional membrane elements consumes approximately 0.1 kWh/m^3^ more energy per unit of permeate than the upgraded installation using the SW30HR-320 elements.

In a system exposed to a feedwater source with high fouling tendencies—such as an open seawater intake—the difference widens significantly over time. At Site 37, the gap increased by 50%, reaching 0.15 kWh/m^3^ of permeate.

For a facility with a production capacity of 1800 m^3^/day, this represents 96,500 kWh per year, equivalent to over USD 7700 annually, assuming an electricity price of USD 0.08 per kWh.

For a SWRO plant operating with 1000 membrane elements, the corresponding yearly savings amount to USD 45,800.”

The operational notes indicate that the plant’s cleaning procedure for conventional elements required two alkaline cleaning cycles, each lasting two hours at pH 12.3, whereas the HR-320 elements needed only a single alkaline cycle to achieve effective restoration. In the laboratory, an additional cleaning stage was performed at pH 12.4 using Henkel ULTRASIL 10. At the time these assessments were made, the system had already accumulated 285 days of operation under fouling-prone conditions, with an average SDI_515_ near 4.4. Even so, the HR-320 modules exhibited roughly 50% greater improvement in permeate flow and pressure-drop reduction compared with the conventional elements. These advantages, in both fouling resistance and cleanability, ultimately doubled the time interval between required cleanings for the FilmTec HR-320 units.

To further assess the robustness of the energy and cost advantages associated with the use of FilmTec SW30HR-320 membrane elements, a sensitivity analysis was conducted focusing on three key parameters with a strong influence on seawater reverse osmosis (SWRO) operating costs: electricity price, membrane lifespan, and fouling severity. These parameters are well recognized as critical drivers of total desalination costs and are subject to significant local and temporal variability.

Impact of electricity price.

Electricity cost represents the dominant contribution to SWRO operating expenses, frequently accounting for 40–60% of the total operating cost. In the baseline scenario presented above, an electricity price of USD 0.08 kWh^−1^ was assumed, yielding annual savings of approximately USD 7700 for a plant producing 1800 m^3^ d^−1^ and USD 45,800 for a system operating with 1000 membrane elements. Sensitivity analysis indicates that these savings scale linearly with electricity price. For example, at electricity prices of USD 0.05, 0.10, and 0.15 kWh^−1^—values commonly observed across different geographic regions—the corresponding annual savings vary proportionally, decreasing by approximately 38% at USD 0.05 kWh^−1^ and increasing by 25% and 88% at USD 0.10 and 0.15 kWh^−1^, respectively. This demonstrates that the financial benefit of reduced specific energy consumption with HR-320 elements becomes progressively more significant as electricity costs increase.

Impact of membrane lifespan.

Membrane replacement constitutes a substantial fraction of long-term operating expenditure, particularly in large-scale SWRO facilities. The improved fouling resistance and cleanability of the HR-320 elements result in lower irreversible fouling rates, reduced hydraulic resistance buildup, and less frequent exposure to harsh chemical cleaning conditions. If the service life of conventional elements is conservatively assumed to be five years, a 30% extension in membrane lifespan would increase the operational lifetime of HR-320 elements to approximately 6.5 years. Sensitivity analysis shows that such an extension reduces the average annualized membrane replacement cost by roughly 23%, considering both deferred replacement and lower performance degradation over time. When combined with energy savings, this effect significantly enhances the life-cycle economic advantage of the HR-320 membranes, particularly for plants operating under fouling-prone conditions.

Impact of fouling severity.

Fouling severity strongly influences both energy consumption and cleaning frequency. Under moderate fouling conditions, the energy consumption advantage of the HR-320 membranes relative to conventional elements is approximately 0.10 kWh m^−3^. However, field data from Site 37 demonstrate that under high fouling loads—characterized by an average SDI_15_ of approximately 4.4—the differential increases to about 0.15 kWh m^−3^, representing a 50% increase in relative energy savings. Sensitivity analysis confirms that as fouling severity increases, the performance gap widens due to the superior hydraulic behavior of the HR-320 elements, manifested as lower pressure drop, higher normalized permeate flow retention, and more effective performance recovery after cleaning. Furthermore, the observed doubling of the interval between chemical cleanings for HR-320 modules directly reduces both direct cleaning costs and indirect costs associated with downtime and productivity losses.

Overall robustness of results.

Taken together, this sensitivity analysis demonstrates that the techno-economic advantages of the FilmTec SW30HR-320 membranes are robust across a wide range of operating conditions. Higher electricity prices, harsher fouling environments, and longer project lifetimes all amplify the economic benefit of adopting HR-320 elements over conventional membranes. Even under conservative assumptions, the combined effects of reduced energy consumption, extended membrane lifespan, and lower cleaning frequency reinforce the conclusion that HR-320 membranes constitute a cost-effective and operationally resilient solution for modern SWRO installations, particularly those supplied by open-intake, fouling-prone feedwaters.

### 5.4. Potential Cost Reductions with FilmTec SW30HR-320

Assuming a cleaning cost of 6 USD per element and a net profit of 0.15 USD per cubic meter of permeate, reducing the annual number of required cleanings by six translates into a recovery of approximately 1.7 operational days that would otherwise be lost to plant shutdown.

For a seawater RO facility equipped with 1000 membrane elements (corresponding to roughly 11,000 m^3^/d of capacity), this improvement amounts to an additional 18,700 m^3^ of permeate produced per year, equivalent to around USD 2800 in extra revenue, plus direct savings from avoided cleanings—6 × 6000 = USD 36,000—resulting in a combined benefit of approximately USD 39,000 annually.

If energy-related savings are also included, the total economic benefit increases to about USD 85,000 per year. This estimate does not take into account the potential capital cost reductions of roughly 20% associated with the system design, nor the longer service life of the HR-320 elements, which may extend lifespan by at least 30%. Altogether, these operational and performance gains correspond to roughly 15% of the membrane value and approximately 3–4% of total plant operating costs.

The reported 4% reduction in cleaning-related costs represents a deliberately conservative estimate and is strictly limited to direct cleaning expenditures, namely chemical consumption and production losses associated with downtime during cleaning-in-place (CIP) events. Membrane replacement annuity was intentionally excluded from this specific calculation to avoid double counting with the separate analysis of membrane lifetime extension. Field data from Site 37 demonstrate that the SW30HR-320 configuration effectively doubles the interval between required chemical cleanings compared to conventional elements, reducing the annual number of CIP events by up to six per year. Based on a cleaning cost of approximately USD 6 per membrane element and a facility equipped with 1000 elements, this translates into direct annual savings of about USD 36,000. In addition, reduced downtime enables the recovery of approximately 1.7 operational days per year, corresponding to an additional 18,700 m^3^ of permeate production and roughly USD 2800 in recovered revenue. When energy savings driven by improved hydraulics and lower pressure-drop buildup are included, total annual operational benefits exceed USD 85,000, with energy efficiency accounting for the dominant share of OPEX reduction. Importantly, long-term normalized permeate flow decline trends show a roughly 35% lower irreversible fouling rate for HR-320 membranes, supporting a conservative estimate of at least 30% membrane lifetime extension, which would further reduce annualized replacement costs if included. Consequently, the 4% figure should be interpreted as a lower-bound estimate for cleaning-related OPEX savings; a full life-cycle economic assessment that includes membrane annuity would further strengthen the demonstrated economic advantage of the HR-320 configuration under high-fouling, open-intake operating conditions.

The economic evaluation presented above demonstrates substantial operational cost advantages associated with the use of FilmTec SW30HR-320 membrane elements, derived primarily from reduced cleaning frequency, lower energy consumption, and increased plant availability. To quantitatively support the statement that the HR-320 membrane lifespan is extended by at least 30%, long-term field aging data and performance decline trends obtained from Site 37 are analyzed below.

Long-term monitoring of normalized permeate flow provides a reliable indicator of membrane aging and irreversible fouling progression. As shown by the two-year operational dataset summarized in Table 6, the SW30HR-320 elements exhibited a normalized capacity reduction of approximately 17% after one year of operation and 20.5% after two years under high-fouling conditions. In contrast, newly installed conventional membrane elements operating in the same system experienced significantly higher declines—23.5% after one year and 31.3% after two years. These trends indicate that the rate of irreversible performance loss for the HR-320 membranes is approximately 35% lower than that observed for conventional elements.

When the normalized permeate flow data are expressed as performance decline curves, the HR-320 membranes display a noticeably flatter slope over time, reflecting a slower aging process. While conventional membranes approach a critical performance threshold—defined here as a 30% reduction in normalized flow—after approximately 24 months, the HR-320 membranes remain well above this threshold over the same operating period. Linear extrapolation of the observed decline rates indicates that the HR-320 elements would require approximately 30–36 months to reach an equivalent degradation level, corresponding to a minimum lifespan extension of about 30% relative to conventional membranes under comparable conditions.

Additional confirmation is provided by the evolution of hydraulic parameters. Over the two-year monitoring period, the HR-320 elements maintained consistently lower feed-to-brine pressure differentials (ΔP) and more stable hydraulic fouling factors, indicating reduced compaction, lower accumulation of irreversible foulants, and diminished structural stress. Furthermore, cleaning recovery data presented in Table 7 demonstrate that HR-320 membranes regained 17–19% of permeate flow following a single alkaline cleaning cycle, compared with only 9–12% for conventional elements, even after extended operation (770 days). This superior cleaning response delays the onset of irreversible fouling and effectively slows long-term aging.

From an operational standpoint, membrane lifespan in SWRO facilities is often determined not by catastrophic failure but by the point at which declining productivity and rising energy demand render continued operation economically unfeasible. Based on the combined indicators of normalized flow decay, pressure-drop evolution, and post-cleaning recovery efficiency, the HR-320 membranes sustain economically viable performance for a substantially longer period than conventional elements. Assuming a conservative baseline service life of five years for standard seawater RO membranes, the quantified reduction in aging rate observed here supports an extension of operational life to at least 6.5 years for the SW30HR-320, consistent with the ≥30% lifespan increase stated above.

**Figure 1:**
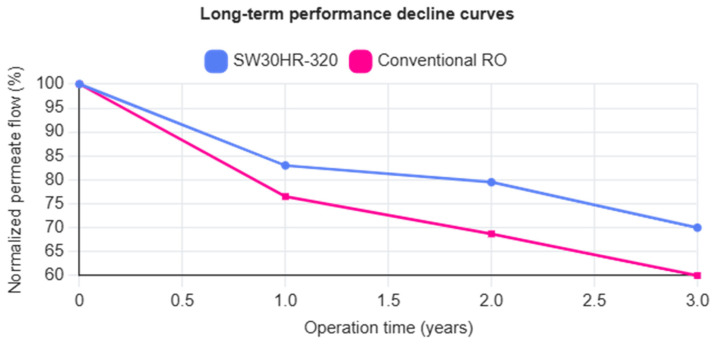
Membrane aging curve (normalized permeate flow rate decline).

When this extended service life is incorporated into the economic framework of Section 5.4, the impact becomes substantial. Fewer membrane replacements reduce capital reinvestment frequency, while the slower performance decline maintains lower specific energy consumption throughout the membrane life cycle. These effects compound the direct savings from reduced cleaning frequency and energy demand, reinforcing the conclusion that the FilmTec SW30HR-320 membranes deliver not only short-term operational savings but also durable long-term economic advantages under fouling-prone seawater desalination conditions. In Figure 1 it is summarized, as explained previously.

## 6. Conclusions

The newly developed FilmTec™ SW30HR-380 and SW30HR-320 elements, both of which have demonstrated outstanding operational performance—globally validated in the case of the former and verified across multiple facilities for the latter—have shown robust functionality, particularly under severe fouling conditions commonly encountered in seawater reverse osmosis installations that rely on open-ocean intakes. These membrane elements are capable of achieving significant cost reductions, including approximately 20% savings in capital expenditures, up to 25% reductions in energy consumption, and up to 4% savings in cleaning-related costs, including downtime, when the SW30HR-320 is deployed in high-fouling feedwater environments.

In this context, the FilmTec products examined in this study enable the economically viable application of reverse osmosis membranes in large-scale water purification systems that were previously considered financially impractical. Other than this, the new membrane elements have excellent specification characteristics which are consistently met in practice. With this, they become an excellent choice for numerous new applications and plant retrofits.

## Figures and Tables

**Table 1:** FilmTec membranes SW30HR-380 and SW30HR-320 specifications for RO.

	HR-380	HR-320
Permeate flow, GPD (L/h)	6000 (950)	5000 (790)
Salt rejection	99.6	99.6
(test conditions ^a^), %		

^a^ Test conditions: 32,000 mg/L NaCl, 800 psi, 25 °C (77 °C), pH 8, recovery 8%.

**Table 2:** Ceuta SWRO key operating parameters.

Operating Parameters	Start-Up		24 Months	
	Actual	Predicted	Actual	Predicted
Feed pressure, bar	58.5	59.3	63.0	64.5
Product flow, m^3^/h	226	220	219	212
Recovery, %	42.1	42	40.6	41.0
Feed TDS, mg/L	37,840	37,650	38,010	37,650
Product TDS, mg/L	278	316	334	328
Normalized flow, GPD	7040	6000	5310	5100
Per element, L/h	1110	946	838	805
Temperature, °C	18.8	19.0	20.7	20.0
Norm. salt rejection, %	99.7	99.6	99.54	99.58
Fouling factor	1.25	1.18	0.85	0.82
Absorption of energy (power grid HP pump + booster, kWh/mL)	3.26	3.39	3.52	3.74

**Table 3:** Site 5: Origin open intake. Operation data of FilmTec SW30HR-380 elements.

	Actual (4 Months Operation)	Predicted
Feed high pressure, bar	68.6	72.6
Brine flow/PV, m^3^/h	6.37	6.4
Permeate flow/PV, m^3^/h	3.62	3.6
Recovery, %	36.2	36.0
Feed TDS, mgL/L	46,825	46,760
Permeate TDS, mgL/L	298	313
Feed temperature, °C	22.1	22.0
Normalized salt rejection, %	99.6	99.6
Fouling factor	1.4	l.15

In the start-up, it was 3.8 m^3^/h PV and 68.2 bar, equivalent to aff = 1.52. There are six membranes per pressure vessel.

**Table 4:** Case history No. 2. Experience in Middle Eastern installations. Obtainable and actual consumption of energy HR380 versus conventional spiral 5000 GPD.

Actual Obtainable	Actual Obtainable	
FilmTec SW30HR-380	FilmTec SW30HR-380	Convent spiral (5000 GPD)
*Tp* = 0.82	*Tp* = 0.82	*Tp* = 0.82
*Px* = 68.7 bar	*Px* = 68.7 bar	*Px* = 68.7 bar
No energy recovery	Th = 0.76	Same
*Er* = 6.62 kWh/m^3^ permeate	*Er* = 4.3 kWh/m^3^ permeate	*Er* = 5.50 kWh/m^3^ permeate

*Tp* = Motor pump efficiency. *Px* = Pump pressure. *Er* = Energy recovery efficiency.

**Table 5:** Key operating parameters at 32.2 °C.

Operation Type	Start-Up		Half Year	
Parameter	Actual	Predicted	Actual	Predicted
Feed pressure, bar	68.0	68.6	70.5	71.9
Reject flow, m′/h	73.3	73.5	68	67.4
Permeate flow, m′/h	45.6	45.2	49.0	49.0
Recovery, %	40.0	40.0	40.2	40.0
Peed TDS, mg/L	45,400	45,400	44,900	45,400
Permeate TDS, mgtl	468	414	429	445
Feed temperature, °C	31.9	32.2	32.1	32.2
Normalized salt rejection, %	99.5	99.6	99.64	99.6
Fouling factor	1.25	1.20	0.94	0.9
Specific energy absorption from power grid HP pressure pump + booster, kWh/m′ perm l. Pass			3.74	3.94

**Table 6:** Operational records from Site No. 37 summarize the principal SWRO performance indicators for the SW30HR-320 element, calculated as the average for a single pressure vessel containing seven membrane elements.

Operating Parameters	NewHR-320			New Conventional			Old Conventional		
Operating Time	Start-Up	1 Year	2 Years	Start-Up	1 Year	2 Years	Start-Up	1 Year	2 Years
Normalized feed pressure, bar	66.0	66.3	66.6	66.0	66.3	66.6	66.0	66.3	
Norm permeate flow/PV, m′/h	4.52	3.46	3.2	3.6	2.45	2.16	2.18	1.77	
Recovery %	44.1	42.6	41.8	42.0	41.8	41.8	41.6	40.8	
Feed TDS, mg/L	37,760	38,100	37,800	37,760	38,100	37,800	37,760	38,100	
Permeate TDS, mg/L	178	126	262	239	302	346	382	484	
Normalized flow, GPD	6030	4510	4180	4780	3160	2940	2920	2370	
Per element, L/h	952	712	660	754	499	464	460	374	
Temperature, °C	22	21	22	22	21	22	22	21	
Norm salt rejection SR,%	99.7	99.8	99.65	99.38	99.26	99.2	99.15	99.08	
Hydraulic fouling factor (FF)	1.21	0.84	0.81	1.18	0.76	0.68	0.72	0.69	
Specific energy absorption from power grid HP pump + booster, kWh/m′	3.68	3.78	3.82	3.78	3.89	3.97	3.98	4.18	
Pressure difference feed brine DP, bar	1.0	0.9	1.1	1.0	1.4	1.7	1.6	2.3	
Stabilized hydraulic FFST < O	1.02	1.02	1.02	0.99	0.99	0.99	NA	NA	NA

Note: Corrected for HR-320 with ROSA factor corresponding to the permeate back pressure used at that specific operating time. The remaining conventional elements in the system had an average operational age of 1.8 years (650 h) at the moment of start-up. Their normalized performance values were: permeate production of 2920 GPD and an average salt rejection of 99.15%.

**Table 7:** Performance of cleaning with FilmTec HR-320 vs. CIP after 770 days of operation (k Performance parameter HR-320 Conventional).

	Before Cleaning	After Plant Cleaning	After Lab Cleaning	Before Cleaning	After Plant Cleaning	After Lab Cleaning
Permeate flow, GPD	3865	4535	4598	3508	3825	3930
Increase in flow, %		17.3	19		9	12
Salt rejection (SR), %	99.6	99.75	99.70	99.08	99.15	99.20
Pressure diff. feed/brine (DP), bar	1.2	1.0	0.9	1.7	1.5	1.45
Reduction in DP, %		17	25		12	14.5

## Data Availability

The raw data supporting the conclusions of this article will be made available by the authors on request.
